# REASONS FOR GRANDFAMILY FORMATION: CHANGING PATTERNS OVER 15 YEARS

**DOI:** 10.1093/geroni/igz038.2498

**Published:** 2019-11-08

**Authors:** McKenzie Wallace, Alexandra B Jeanblanc, Carol M Musil

**Affiliations:** Case Western Reserve University, Cleveland, Ohio, United States

## Abstract

As the number of grandparent-headed households continues to rise, it is vital to examine the changing impetus for the formation of grandfamilies. We compared the reasons for caregiving of custodial grandmothers from a current study (2017-2019: N=236) to a previous study (2002-2003: N=183) to determine whether reasons for caregiving have shifted over the 15 year period. Participants were asked to describe the reason for caregiving in an open ended question. The responses were coded into 17 categories. The percentage of participants who described each category as being a reason of caregiving were compared to determine any changes in reasons for caregiving. In both samples drug use and abandonment were the top reasons for caregiving. While abandonment shows no change (30% vs 28%), the current study shows a significant increase in drug use (40% vs 21%). The frequency at which child services was involved increased from 10% in the original sample to 28% in the current sample. Instances of violence in the home significantly increased from the original study (0.5%) to the current study (12%). Other reasons, child abuse, death of a parent, financial strain, parental health, and relationship strife, remained similar between the two samples. These data may represent an increase in family life events, but also greater willingness to disclose disruptive family life events. The substantial increase in drug use and violence as reasons for grandparents to become caregivers is concerning and suggests critical direction for clinical practice, research, and policy change to support grandparent caregivers.

